# (*S*)-Ethyl 2-[4-(6-chloro­quinoxalin-2-yl­oxy)phen­oxy]propanoate

**DOI:** 10.1107/S1600536809030074

**Published:** 2009-08-08

**Authors:** Jun Hu, Guo-song Chen, Li-hua Guo, Ji-kui Wang, Yan-hua Xu

**Affiliations:** a Department of Applied Chemistry, College of Science, Nanjing University of Technology, Nanjing 210009, People’s Republic of China; b Department of Safety Engineering, College of Urban Construction and Safety & Environmental Engineering, Nanjing University of Technology, Nanjing 210009, People’s Republic of China

## Abstract

In the mol­ecule of the title compound, C_19_H_17_ClN_2_O_4_, the quinoxaline ring system is planar [maximum deviation = 0.013 (3) Å] and oriented at a dihedral angle of 80.18 (3)° with respect to the benzene ring. In the crystal structure, inter­molecular C—H⋯N inter­actions link mol­ecules into chains. π–π contacts between the quinoxaline systems [centroid–centroid distance = 3.654 (1) Å] may further stabilize the structure.

## Related literature

The title compound has potent selective herbicidal activity against annual and perennial grass weeds, see: Sakata *et al.* (1985 ▶). For bond-length data, see: Allen *et al.* (1987 ▶).
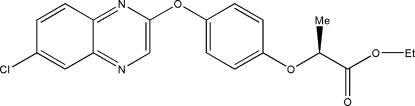


## Experimental

### 

#### Crystal data

C_19_H_17_ClN_2_O_4_
*M* *_r_* = 372.80
Monoclinic, 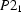

*a* = 9.970 (2) Å
*b* = 4.4760 (9) Å
*c* = 20.450 (4) Å
β = 94.54 (3)°
*V* = 909.7 (3) Å^3^
*Z* = 2
Mo *K*α radiation
μ = 0.24 mm^−1^
*T* = 294 K
0.30 × 0.20 × 0.10 mm

#### Data collection

Enraf–Nonius CAD-4 diffractometer
Absorption correction: ψ scan (North *et al.*, 1968 ▶) *T*_min_ = 0.932, *T*_max_ = 0.977
3762 measured reflections
1898 independent reflections
1254 reflections with *I* > 2σ(*I*)
*R*_int_ = 0.052
3 standard reflections frequency: 120 min intensity decay: 1%

#### Refinement

*R*[*F* ^2^ > 2σ(*F* ^2^)] = 0.050
*wR*(*F* ^2^) = 0.155
*S* = 1.00
1898 reflections
235 parameters
1 restraint
H-atom parameters constrained
Δρ_max_ = 0.17 e Å^−3^
Δρ_min_ = −0.20 e Å^−3^
Absolute structure: Flack (1983 ▶), 932 Friedel pairs
Flack parameter: −0.02 (18)

### 

Data collection: *CAD-4 Software* (Enraf–Nonius, 1985 ▶); cell refinement: *CAD-4 Software*; data reduction: *XCAD4* (Harms & Wocadlo, 1995 ▶); program(s) used to solve structure: *SHELXS97* (Sheldrick, 2008 ▶); program(s) used to refine structure: *SHELXL97* (Sheldrick, 2008 ▶); molecular graphics: *SHELXTL* (Sheldrick, 2008 ▶); software used to prepare material for publication: *SHELXTL*.

## Supplementary Material

Crystal structure: contains datablocks I, Il. DOI: 10.1107/S1600536809030074/hk2712sup1.cif

Structure factors: contains datablocks I. DOI: 10.1107/S1600536809030074/hk2712Isup2.hkl

Additional supplementary materials:  crystallographic information; 3D view; checkCIF report

## Figures and Tables

**Table 1:** Hydrogen-bond geometry (Å, °)

*D*—H⋯*A*	*D*—H	H⋯*A*	*D*⋯*A*	*D*—H⋯*A*
C19—H19*A*⋯N1^i^	0.93	2.57	3.396 (7)	149

Symmetry code: (i) 
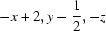
.

**Fig. 1.**
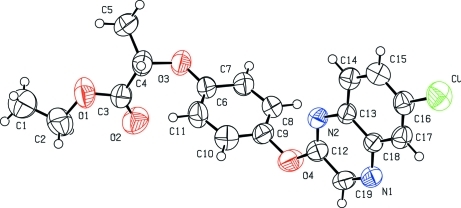
The molecular structure of the title molecule, with the atom-numbering scheme. Displacement ellipsoids are drawn at the 50% probability level.

**Fig. 2.**
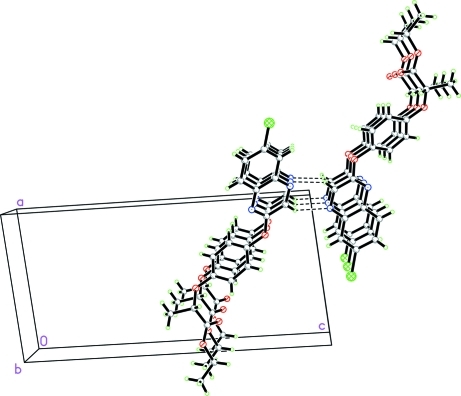
A partial packing diagram of the title compound. Hydrogen bonds are shown as dashed lines.

## supplementary crystallographic information

### Comment

The title compound has a potent selective herbicidal activity against annual and perennial grass weeds (Sakata *et al.*, 1985). We report herein its crystal structure.

In the molecule of the title compound (Fig. 1), the bond lengths (Allen *et al.*,  1987) and angles are within normal ranges. Rings A (C6-C11), B (C13-C18) and C (N1/N2/C12/C13/C18/C19) are, of course, planar, and they are oriented at dihedral angles of A/B = 80.21 (3), A/C = 80.07 (3) and B/C = 0.66 (3) °. The quinoxaline ring system is planar with a maximum deviation of -0.013 (3) Å for atom N1.

In the crystal structure, intermolecular C-H···N interactions link the molecules into chains (Fig. 2), in which they may be effective in the stabilization of the structure. The π–π contact between the quinoxaline rings, Cg2—Cg3^i^ [symmetry code: (i) x, y - 1, z, where Cg2 and Cg3 are centroids of the rings B (C13-C18) and C (N1/N2/C12/C13/C18/C19), respectively] may further stabilize the structure, with centroid-centroid distance of 3.654 (1) Å.

### Experimental

For the preparation of the title compound, thionyl chloride (3.7 ml, 50 mmol) was added in dropwise to (*S*)-2-(4-(6-chloroquinoxalin-2-yloxy)phenoxy)propanoate acid (3.72 g, 10 mmol) in an ice bath (263 K). After refluxing for 5 h, the mixture was cooled to room temperature, and excess thionyl chloride was removed by reduced pressure distillation. Then, the residue was dissolved in a solution of ethanol (4.9 ml, 80 mmol) and pyridine (2.5 ml, 30 mmol). The solid residue was extracted with hexane (40 ml) and hexane was distilled off. Crystals suitable for X-ray analysis were formed after 8 d in ethyl acetate by slow evaporation at room temperature.

### Refinement

H atoms were positioned geometrically with C-H = 0.93, 0.98, 0.97 and 0.96 Å, for aromatic, methine, methylene and methyl H atoms, respectively, and constrained to ride on their parent atoms, with U_iso_(H) = xU_eq_(C), where x = 1.5 for methyl H and x = 1.2 for aromatic H atoms.

### Crystal data

**Table:**

C_19_H_17_ClN_2_O_4_	*F*(000) = 388
*M**_r_* = 372.80	*D*_x_ = 1.361 Mg m^−^^3^
Monoclinic, *P*2_1_	Melting point: 350 K
Hall symbol: P 2yb	Mo *K*α radiation, λ = 0.71073 Å
*a* = 9.970 (2) Å	Cell parameters from 25 reflections
*b* = 4.4760 (9) Å	θ = 9–12°
*c* = 20.450 (4) Å	µ = 0.24 mm^−^^1^
β = 94.54 (3)°	*T* = 294 K
*V* = 909.7 (3) Å^3^	Needle, colorless
*Z* = 2	0.30 × 0.20 × 0.10 mm

### Data collection

**Table:**

Enraf–Nonius CAD-4 diffractometer	1254 reflections with *I* > 2σ(*I*)
Radiation source: fine-focus sealed tube	*R*_int_ = 0.052
graphite	θ_max_ = 25.4°, θ_min_ = 2.0°
ω/2θ scans	*h* = −11→11
Absorption correction: ψ scan (North *et al.*, 1968)	*k* = −5→0
*T*_min_ = 0.932, *T*_max_ = 0.977	*l* = −24→24
3762 measured reflections	3 standard reflections every 120 min
1898 independent reflections	intensity decay: 1%

### Refinement

**Table:**

Refinement on *F*^2^	Secondary atom site location: difference Fourier map
Least-squares matrix: full	Hydrogen site location: inferred from neighbouring sites
*R*[*F*^2^ > 2σ(*F*^2^)] = 0.050	H-atom parameters constrained
*wR*(*F*^2^) = 0.155	*w* = 1/[σ^2^(*F*_o_^2^) + (0.085*P*)^2^] where *P* = (*F*_o_^2^ + 2*F*_c_^2^)/3
*S* = 1.00	(Δ/σ)_max_ < 0.001
1898 reflections	Δρ_max_ = 0.17 e Å^−^^3^
235 parameters	Δρ_min_ = −0.19 e Å^−^^3^
1 restraint	Absolute structure: Flack (1983), 932 Friedel pairs
Primary atom site location: structure-invariant direct methods	Flack parameter: −0.02 (18)

### Special details

**Table:**

Experimental. Refinement of F^2^ against ALL reflections. The weighted *R*-factor *wR* and goodness of fit S are based on F^2^, conventional *R*-factors *R* are based on F, with F set to zero for negative F^2^. The threshold expression of F^2^ > 2sigma(F^2^) is used only for calculating *R*-factors(gt) *etc*. and is not relevant to the choice of reflections for refinement. *R*-factors based on F^2^ are statistically about twice as large as those based on F, and R– factors based on ALL data will be even larger.
Geometry. All e.s.d.'s (except the e.s.d. in the dihedral angle between two l.s. planes) are estimated using the full covariance matrix. The cell e.s.d.'s are taken into account individually in the estimation of e.s.d.'s in distances, angles and torsion angles; correlations between e.s.d.'s in cell parameters are only used when they are defined by crystal symmetry. An approximate (isotropic) treatment of cell e.s.d.'s is used for estimating e.s.d.'s involving l.s. planes.
Refinement. Refinement of *F*^2^ against ALL reflections. The weighted *R*-factor *wR* and goodness of fit *S* are based on *F*^2^, conventional *R*-factors *R* are based on *F*, with *F* set to zero for negative *F*^2^. The threshold expression of *F*^2^ > σ(*F*^2^) is used only for calculating *R*-factors(gt) *etc*. and is not relevant to the choice of reflections for refinement. *R*-factors based on *F*^2^ are statistically about twice as large as those based on *F*, and *R*- factors based on ALL data will be even larger.

### Fractional atomic coordinates and isotropic or equivalent isotropic displacement parameters (Å^2^)

**Table:**

	*x*	*y*	*z*	*U*_iso_*/*U*_eq_	
Cl	0.50826 (14)	0.9066 (5)	0.08796 (7)	0.0921 (6)	
O1	1.8615 (3)	0.4503 (12)	0.3878 (2)	0.0925 (14)	
O2	1.7523 (4)	0.0871 (12)	0.3367 (2)	0.0956 (14)	
O3	1.5198 (3)	0.2389 (10)	0.38949 (16)	0.0730 (11)	
O4	1.2147 (3)	0.1148 (12)	0.15458 (16)	0.0838 (13)	
N1	0.9132 (4)	0.2496 (12)	0.05649 (17)	0.0616 (11)	
N2	1.0378 (4)	0.4196 (12)	0.18081 (17)	0.0563 (10)	
C1	2.0926 (5)	0.474 (2)	0.4119 (4)	0.131 (3)	
H1B	2.1793	0.4138	0.3993	0.196*	
H1C	2.0803	0.3998	0.4551	0.196*	
H1D	2.0871	0.6882	0.4120	0.196*	
C2	1.9882 (5)	0.353 (2)	0.3656 (3)	0.095 (2)	
H2B	1.9988	0.4282	0.3218	0.115*	
H2C	1.9928	0.1370	0.3648	0.115*	
C3	1.7536 (5)	0.3000 (15)	0.3698 (3)	0.0657 (15)	
C4	1.6313 (4)	0.4380 (16)	0.3988 (2)	0.0710 (15)	
H4A	1.6092	0.6308	0.3779	0.085*	
C5	1.6551 (6)	0.476 (2)	0.4726 (3)	0.107 (3)	
H5A	1.5771	0.5645	0.4893	0.161*	
H5B	1.7316	0.6029	0.4824	0.161*	
H5C	1.6716	0.2841	0.4927	0.161*	
C6	1.4468 (4)	0.2317 (14)	0.3298 (2)	0.0582 (13)	
C7	1.3386 (5)	0.0408 (14)	0.3263 (2)	0.0656 (14)	
H7A	1.3203	−0.0682	0.3633	0.079*	
C8	1.2570 (5)	0.0074 (15)	0.2696 (2)	0.0715 (17)	
H8A	1.1837	−0.1217	0.2677	0.086*	
C9	1.2861 (5)	0.1681 (15)	0.2160 (2)	0.0637 (15)	
C10	1.3937 (5)	0.3564 (15)	0.2181 (3)	0.0720 (15)	
H10A	1.4119	0.4639	0.1808	0.086*	
C11	1.4758 (5)	0.3883 (16)	0.2752 (3)	0.0718 (15)	
H11A	1.5500	0.5147	0.2765	0.086*	
C12	1.0919 (5)	0.2395 (14)	0.1409 (2)	0.0612 (13)	
C13	0.9127 (4)	0.5295 (12)	0.1580 (2)	0.0517 (12)	
C14	0.8466 (5)	0.7268 (13)	0.1979 (2)	0.0586 (13)	
H14A	0.8859	0.7811	0.2389	0.070*	
C15	0.7228 (5)	0.8395 (14)	0.1757 (2)	0.0658 (15)	
H15A	0.6783	0.9715	0.2017	0.079*	
C16	0.6646 (5)	0.7562 (14)	0.1148 (2)	0.0626 (14)	
C17	0.7261 (5)	0.5646 (14)	0.0749 (2)	0.0614 (14)	
H17A	0.6852	0.5136	0.0340	0.074*	
C18	0.8516 (4)	0.4457 (12)	0.0964 (2)	0.0509 (12)	
C19	1.0293 (5)	0.1538 (15)	0.0790 (2)	0.0646 (15)	
H19A	1.0746	0.0216	0.0534	0.078*	

### Atomic displacement parameters (Å^2^)

**Table:**

	*U*^11^	*U*^22^	*U*^33^	*U*^12^	*U*^13^	*U*^23^
Cl	0.0714 (9)	0.1061 (14)	0.0958 (11)	0.0193 (10)	−0.0124 (7)	0.0154 (11)
O1	0.056 (2)	0.079 (3)	0.141 (3)	−0.003 (2)	0.003 (2)	−0.029 (3)
O2	0.092 (3)	0.083 (3)	0.111 (3)	0.010 (3)	0.002 (2)	−0.037 (3)
O3	0.063 (2)	0.087 (3)	0.067 (2)	−0.018 (2)	−0.0070 (16)	0.000 (2)
O4	0.065 (2)	0.115 (4)	0.070 (2)	0.028 (3)	−0.0046 (17)	−0.022 (2)
N1	0.061 (2)	0.071 (3)	0.051 (2)	−0.001 (3)	−0.0001 (18)	0.002 (2)
N2	0.054 (2)	0.066 (3)	0.049 (2)	0.001 (2)	−0.0021 (16)	−0.001 (2)
C1	0.062 (4)	0.123 (8)	0.205 (8)	0.012 (5)	−0.004 (4)	−0.024 (7)
C2	0.066 (3)	0.099 (5)	0.123 (5)	0.013 (4)	0.016 (3)	0.007 (5)
C3	0.061 (3)	0.067 (4)	0.068 (3)	0.001 (3)	−0.003 (2)	0.000 (3)
C4	0.055 (3)	0.067 (4)	0.089 (4)	−0.005 (3)	−0.004 (2)	−0.014 (3)
C5	0.078 (4)	0.156 (8)	0.087 (4)	−0.006 (5)	0.001 (3)	−0.055 (5)
C6	0.048 (2)	0.064 (3)	0.063 (3)	−0.002 (3)	0.004 (2)	−0.006 (3)
C7	0.058 (3)	0.074 (4)	0.064 (3)	−0.009 (3)	0.004 (2)	−0.001 (3)
C8	0.054 (3)	0.091 (5)	0.070 (3)	−0.010 (3)	0.009 (2)	−0.013 (3)
C9	0.051 (3)	0.077 (4)	0.062 (3)	0.014 (3)	−0.004 (2)	−0.016 (3)
C10	0.073 (3)	0.070 (4)	0.072 (3)	0.002 (3)	0.002 (3)	0.008 (3)
C11	0.061 (3)	0.074 (4)	0.080 (3)	−0.017 (3)	−0.002 (2)	0.011 (4)
C12	0.060 (3)	0.070 (4)	0.053 (3)	0.000 (3)	0.002 (2)	0.002 (3)
C13	0.052 (2)	0.056 (3)	0.047 (2)	−0.007 (2)	−0.0007 (19)	0.005 (2)
C14	0.061 (3)	0.057 (3)	0.056 (3)	−0.002 (3)	−0.001 (2)	0.004 (3)
C15	0.069 (3)	0.062 (4)	0.067 (3)	0.007 (3)	0.010 (2)	0.004 (3)
C16	0.055 (3)	0.066 (4)	0.065 (3)	−0.001 (3)	−0.002 (2)	0.015 (3)
C17	0.065 (3)	0.069 (4)	0.049 (2)	−0.006 (3)	−0.010 (2)	0.011 (3)
C18	0.056 (2)	0.051 (3)	0.045 (2)	−0.010 (3)	0.0043 (19)	0.003 (2)
C19	0.072 (3)	0.076 (4)	0.047 (2)	0.001 (3)	0.006 (2)	−0.010 (3)

### Geometric parameters (Å, °)

**Table:**

Cl—C16	1.746 (5)	C5—H5C	0.9600
O1—C2	1.443 (6)	C6—C11	1.370 (7)
O1—C3	1.298 (7)	C6—C7	1.373 (7)
O2—C3	1.168 (7)	C7—C8	1.372 (6)
O3—C4	1.426 (6)	C7—H7A	0.9300
O3—C6	1.371 (5)	C8—C9	1.361 (8)
O4—C9	1.414 (6)	C8—H8A	0.9300
O4—C12	1.355 (6)	C9—C10	1.363 (8)
N1—C18	1.375 (6)	C10—C11	1.379 (7)
N1—C19	1.285 (6)	C10—H10A	0.9300
N2—C12	1.294 (7)	C11—H11A	0.9300
N2—C13	1.386 (6)	C12—C19	1.420 (6)
C1—C2	1.455 (9)	C13—C14	1.401 (7)
C1—H1B	0.9600	C13—C18	1.407 (6)
C1—H1C	0.9600	C14—C15	1.376 (7)
C1—H1D	0.9600	C14—H14A	0.9300
C2—H2B	0.9700	C15—C16	1.383 (7)
C2—H2C	0.9700	C15—H15A	0.9300
C3—C4	1.528 (8)	C16—C17	1.362 (7)
C4—C5	1.519 (7)	C17—C18	1.398 (6)
C4—H4A	0.9800	C17—H17A	0.9300
C5—H5A	0.9600	C19—H19A	0.9300
C5—H5B	0.9600		
			
C3—O1—C2	118.8 (5)	C6—C7—H7A	119.2
C6—O3—C4	119.1 (4)	C9—C8—C7	118.3 (5)
C12—O4—C9	119.8 (4)	C9—C8—H8A	120.9
C19—N1—C18	115.6 (4)	C7—C8—H8A	120.9
C12—N2—C13	114.7 (4)	C8—C9—C10	121.3 (5)
C2—C1—H1B	109.5	C8—C9—O4	120.1 (5)
C2—C1—H1C	109.5	C10—C9—O4	118.1 (5)
C2—C1—H1D	109.5	C9—C10—C11	120.2 (5)
H1B—C1—H1C	109.5	C9—C10—H10A	119.9
H1B—C1—H1D	109.5	C11—C10—H10A	119.9
H1C—C1—H1D	109.5	C6—C11—C10	119.2 (5)
O1—C2—C1	106.4 (6)	C6—C11—H11A	120.4
O1—C2—H2B	110.4	C10—C11—H11A	120.4
C1—C2—H2B	110.4	N2—C12—O4	122.8 (4)
O1—C2—H2C	110.4	N2—C12—C19	123.7 (5)
C1—C2—H2C	110.4	O4—C12—C19	113.5 (5)
H2B—C2—H2C	108.6	N2—C13—C14	118.7 (4)
O2—C3—O1	124.0 (6)	N2—C13—C18	121.4 (4)
O2—C3—C4	125.6 (6)	C14—C13—C18	119.9 (4)
O1—C3—C4	110.4 (5)	C15—C14—C13	119.4 (4)
O3—C4—C5	105.1 (5)	C15—C14—H14A	120.3
O3—C4—C3	109.5 (5)	C13—C14—H14A	120.3
C5—C4—C3	111.4 (4)	C14—C15—C16	119.9 (5)
O3—C4—H4A	110.3	C14—C15—H15A	120.1
C5—C4—H4A	110.3	C16—C15—H15A	120.1
C3—C4—H4A	110.3	C17—C16—C15	122.2 (5)
C4—C5—H5A	109.5	C17—C16—Cl	119.2 (4)
C4—C5—H5B	109.5	C15—C16—Cl	118.6 (5)
H5A—C5—H5B	109.5	C16—C17—C18	119.1 (4)
C4—C5—H5C	109.5	C16—C17—H17A	120.4
H5A—C5—H5C	109.5	C18—C17—H17A	120.4
H5B—C5—H5C	109.5	N1—C18—C17	119.2 (4)
C11—C6—O3	125.7 (5)	N1—C18—C13	121.3 (4)
C11—C6—C7	119.5 (5)	C17—C18—C13	119.5 (5)
O3—C6—C7	114.9 (5)	N1—C19—C12	123.3 (5)
C8—C7—C6	121.5 (5)	N1—C19—H19A	118.3
C8—C7—H7A	119.2	C12—C19—H19A	118.3
			
C3—O1—C2—C1	−157.4 (6)	C13—N2—C12—C19	−1.0 (8)
C2—O1—C3—O2	0.7 (9)	C9—O4—C12—N2	3.3 (9)
C2—O1—C3—C4	179.7 (5)	C9—O4—C12—C19	−176.8 (5)
C6—O3—C4—C5	159.6 (5)	C12—N2—C13—C14	−179.9 (5)
C6—O3—C4—C3	−80.7 (6)	C12—N2—C13—C18	0.4 (7)
O2—C3—C4—O3	10.5 (8)	N2—C13—C14—C15	179.5 (5)
O1—C3—C4—O3	−168.5 (4)	C18—C13—C14—C15	−0.8 (7)
O2—C3—C4—C5	126.3 (7)	C13—C14—C15—C16	0.4 (8)
O1—C3—C4—C5	−52.7 (8)	C14—C15—C16—C17	−0.3 (8)
C4—O3—C6—C11	4.3 (8)	C14—C15—C16—Cl	−179.3 (4)
C4—O3—C6—C7	−177.8 (5)	C15—C16—C17—C18	0.6 (8)
C11—C6—C7—C8	−1.3 (9)	Cl—C16—C17—C18	179.7 (4)
O3—C6—C7—C8	−179.3 (5)	C19—N1—C18—C17	178.8 (5)
C6—C7—C8—C9	0.3 (8)	C19—N1—C18—C13	−0.9 (7)
C7—C8—C9—C10	0.4 (8)	C16—C17—C18—N1	179.3 (5)
C7—C8—C9—O4	172.5 (5)	C16—C17—C18—C13	−1.0 (7)
C12—O4—C9—C8	81.4 (7)	N2—C13—C18—N1	0.5 (7)
C12—O4—C9—C10	−106.3 (6)	C14—C13—C18—N1	−179.2 (5)
C8—C9—C10—C11	−0.1 (9)	N2—C13—C18—C17	−179.2 (5)
O4—C9—C10—C11	−172.4 (6)	C14—C13—C18—C17	1.1 (7)
O3—C6—C11—C10	179.3 (5)	C18—N1—C19—C12	0.4 (8)
C7—C6—C11—C10	1.5 (9)	N2—C12—C19—N1	0.6 (9)
C9—C10—C11—C6	−0.9 (9)	O4—C12—C19—N1	−179.2 (5)
C13—N2—C12—O4	178.9 (5)		

### Hydrogen-bond geometry (Å, °)

**Table:**

*D*—H···*A*	*D*—H	H···*A*	*D*···*A*	*D*—H···*A*
C19—H19A···N1^i^	0.93	2.57	3.396 (7)	149

Symmetry codes: (i) −*x*+2, *y*−1/2, −*z*.
